# Re: Comment to “Relationship between exposure to ionizing radiation and mesothelioma risk: A systematic review of the scientific literature and meta‐analysis”: Cancer Med. 2022 Feb;11(3):778‐789. doi: 10.1002/cam4.4436. Epub 2022 Jan 14

**DOI:** 10.1002/cam4.4803

**Published:** 2022-05-10

**Authors:** Giovanni Visci, Carlotta Zunarelli, Paolo Boffetta, Francesco Violante

**Affiliations:** ^1^ Department of Medical and Surgical Sciences University of Bologna Bologna Italy; ^2^ IRCCS S. Orsola University Hospital Bologna Italy; ^3^ Stony Brook Cancer Center Stony Brook University Stony Brook New York USA

To the Editor, Cancer Medicine

Sir,

After the publication of our meta‐analysis entitled, “Relationship between exposure to ionizing radiation and mesothelioma risk: A systematic review of the scientific literature and meta‐analysis”,^1^ we have become aware of an additional study that reported results on mesothelioma mortality among workers exposed to ionizing radiation.

Mumma and coworkers^2^ studied a cohort of 253,632 United States workers who were routinely monitored for external radiation, including 30,724 industrial radiographers (IR) at shipyards, 142,583 workers at nuclear power plants (NPP), and 83,441 IR who had not worked at an NPP or shipyard. The mean cumulative lung dose was 28.6 mGy. In a follow‐up from 1969 through 2011, comprising almost 6.5 million person‐years, 421 deaths from mesothelioma were observed, yielding a standardized mortality ratio (SMR) of 6.10 (95% confidence interval [CI] 5.53–6.71). The increased mortality from mesothelioma was found among shipyards (SMR 9.97; 95% CI 8.50–11.63) and NPP workers (SMR 5.55; 95% CI 4.88–6.29), but not among IR who had not worked in NPP or shipyards (SMR 1.15; 95% CI 0.53–2.19).

When we repeated the meta‐analysis including this additional cohort using the methodology described in our recent report,^1^ we calculated a pooled relative risk (RR) of 4.43 (95% CI 2.77–7.09; Figure 1), compared to a pooled RR of 3.57 (95% CI 2.16–5.89) reported in our study.^1^

**FIGURE 1:**
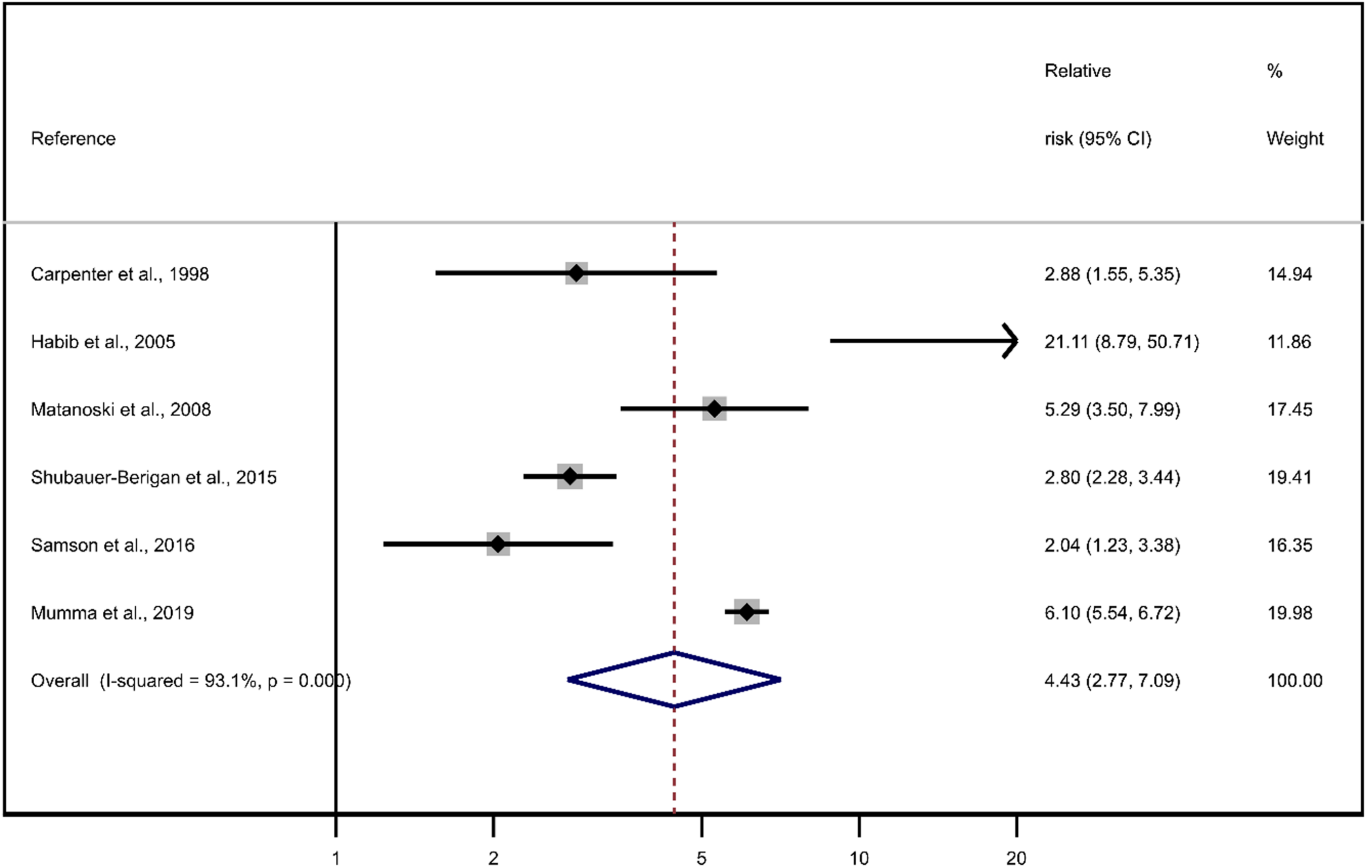
Updated meta‐analysis of studies of workers exposed to ionizing radiation (see^1^ for references to the original studies).

In conclusion, the inclusion of the sixth cohort of workers exposed to ionizing radiation did not modify our previous conclusion that these workers experienced an increased risk of mesothelioma.^1^ However, the fact that in the additional cohort the increased risk was restricted to workers with concomitant potential exposure to asbestos, and not among workers exposed to ionizing radiation but not to asbestos (IR employed outside NPP or shipyard), suggests caution in attributing the excess risk of mesothelioma to occupational exposure to ionizing radiation.

## FUNDING INFORMATION

The study was funded with internal resources of the participating institutions.

## CONFLICT OF INTEREST

The authors declare no conflicts of interest.

## AUTHOR CONTRIBUTIONS

Paolo Boffetta, Giovanni Visci, Carlotta Zunarelli: design of the study. Paolo Boffetta, Francesco Violante: supervision. Paolo Boffetta, Francesco Violante, Giovanni Visci, Carlotta Zunarelli: drafting of the manuscript.

## ETHICS STATEMENT

As this review and meta‐analysis were a retrospective observational study, no questions were asked to the ethics committee.

## Data Availability

All the primary data are available from the first author.
